# Selectivity Control of Oxygen Reduction Reaction over Mesoporous Transition Metal Oxide Catalysts for Electrified Purification Technologies

**DOI:** 10.1021/acsami.3c01196

**Published:** 2023-05-19

**Authors:** Zhixing Wu, Mikhail Vagin, Robert Boyd, Penghui Ding, Oleksandr Pshyk, Grzegorz Greczynski, Magnus Odén, Emma M. Björk

**Affiliations:** † Nanostructured Materials, Department of Physics, Chemistry and Biology (IFM), Linköping University, Linköping SE 58183, Sweden; ‡ Laboratory of Organic Electronics, Department of Science and Technology (ITN), Linköping University, Norrköping SE 60174, Sweden; § Thin Film Physics, Department of Physics, Chemistry and Biology (IFM), Linköping University, Linköping SE 58183, Sweden

**Keywords:** oxygen reduction reaction, oxygen evolution reaction, oxygen on demand, mesoporous oxides, nickel(II) oxide, nickel cobaltite, hydroxyl radical

## Abstract

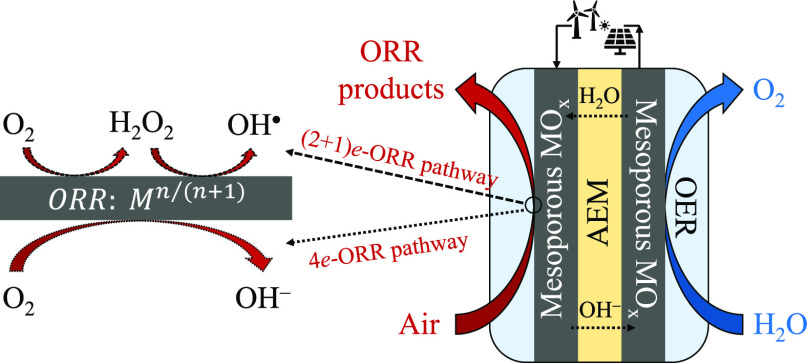

Direct electrification of oxygen-associated reactions contributes to large-scale electrical storage and the launch of the green hydrogen economy. The design of the involved catalysts can mitigate the electrical energy losses and improve the control of the reaction products. We evaluate the effect of the interface composition of electrocatalysts on the efficiency and productivity of the oxygen reduction reaction (ORR) and oxygen evolution reaction (OER), both mechanistically and at device levels. The ORR and OER were benchmarked on mesoporous nickel(II) oxide and nickel cobaltite (NiO and NiCo_2_O_4_, respectively) obtained by a facile template-free hydrothermal synthesis. Physicochemical characterization showed that both NiO and NiCo_2_O_4_ are mesoporous and have a cubic crystal structure with abundant surface hydroxyl species. NiCo_2_O_4_ showed higher electrocatalytic activity in OER and selectivity to water as the terminal product of ORR. On the contrary, ORR over NiO yielded hydroxyl radicals as products of a Fenton-like reaction of H_2_O_2_. The product selectivity in ORR was used to construct two electrolyzers for electrified purification of oxygen and generation of hydroxyl radicals.

## 1 Introduction

**Scheme 1:**
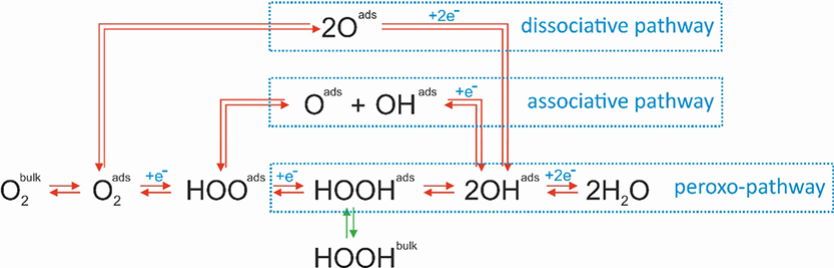
The Mechanistic Landscape of ORR and OER (Left to Right and Right to Left, Respectively) X_ads_ and X_bulk_ imply the species adsorbed and desorbed from the catalyst surface.

The use of abundant feedstocks, such as water and air, in manufacturing of value-added products is important in achieving the UN’s Sustainable Development Goals.^1^ In this context, electrified direct synthesis of chemicals from oxygen in the air is of special interest because it avoids greenhouse gas emissions if the electricity used to drive the synthesis comes from sustainable sources such as solar and wind power. The complexity of multielectronic reaction mechanisms of direct oxygen transformations (Scheme 1), i.e., oxygen reduction reaction (ORR) and oxygen evolution reaction (OER), results in slow reaction kinetics on a general electrified interface hosting heterogeneous electron transfer. Chemisorption of reaction intermediates decreases the activation barriers between elementary steps of the reaction, resulting in the mitigation of electrical losses during the electrosynthesis.

The value of the industrial production of pure oxygen is estimated to be 27 billion USD in 2021 (which is forecasted to have grown by 8% by 2025).^2^ Oxygen production relies mainly on the cryogenic distillation of air, which is a centralized and energy-demanding process. The high reactivity of oxygen is a safety issue, especially when large quantities are stored, transported, and handled, and is thereby associated with significant safety costs. The limitations of a centralized oxygen production infrastructure became obvious as the demand for oxygen for COVID-19 patients increased and the inflexibility of the current production facilities could not meet the acute need for oxygen.^3,4^ On the other hand, water electrolysis produces high purity of oxygen on the anode with hydrogen as the byproduct at the cathode by requiring a minimum cell voltage of 1.23 V.^5^ The oxygen-on-demand technologies intend to provide oxygen on-site for further utilization, which reduces the expenses of safe transportation and storage. One example of an existing oxygen-on-demand technology is the electrochemical oxygen pump based on electrochemical purification of oxygen from air. The oxygen pump utilizes OER from water for production of oxygen via membrane electrolysis with a charge compensation provided by the ORR of incoming air to water.^6^ In this approach, oxygen is not produced from the air used but rather it is synthesized from water. Thus, even heavily contaminated air can be used.

Clean water and sanitation are another goal (Goal 7) among the 17 sustainable development goals. Indeed, the hydroxyl radical (OH·) is a strong oxidant with the highest oxidation capability among the different reactive oxygen species (oxidation potential 2.8 V). Because of its high reactivity and broad applicability, the in situ generated hydroxyl radical can be utilized for purification technologies such as advanced oxidation process for water treatment.^7,8^ Photocatalytic generation of hydroxyl radical has been widely studied via reducing the O_2_ using photocatalysts, e.g., TiO_2_ and ZnO_2_.^9,10^ However, the photocatalytic process has a relatively low degradation efficiency due to the limited adsorbed light i.e., <5% adsorption of UV light.^11^ Electrocatalysis provides a promising approach for OH· generation using O_2_ as feedstock, and interest in this approach has emerged recently. The possibility of in situ generation of the hydroxyl radical by electrified ORR has been reported for a few catalysts such as palladium and titanium oxides.^12,13^ Furthermore, the OH· generated on the FeCoC catalyst via a so-called three-electron ORR pathway has been applied for the removal of ciprofloxacin in half cells.^8^

To reduce energy losses in electrochemical devices, catalysts are often used.^14,15^ Platinum group metals (PGMs) are traditionally used as catalysts for oxygen conversion reactions in acidic and basic media. The drawback is that PGMs are critical raw materials of supply risks, for which there are no easy substitutes.^16^ Instead, inexpensive PGM-free catalysts, e.g., transition metal oxides and hydroxides, can be used to drive oxygen electrosynthesis by OER in alkaline media,^5,17^ with capabilities similar to PGM-based catalysts. Optimization of the catalyst’s surface area and structure by introduction of mesoporosity decreases the electrical losses during OER.^18−20^

Among different intermediates of multielectron ORR (Scheme 1), hydrogen peroxide (H_2_O_2_) is the most stable one in the peroxo-pathway. Hence, hydrogen peroxide will desorb from the catalyst surface into the electrolyte where it can either remain intact or undergo subsequent chemical (electrochemistry-free) postreactions, such as disproportionation to oxygen and water (2H_2_O_2_ → O_2_ + 2H_2_O)^21^ and a Fenton-like reaction (H_2_O_2_ + M^*n*+^ → OH · + M^(*n* + 1)+^ + OH^–^),^22,23^ where M^*n*+^/M^(*n* + 1)+^ are transition metal ions available for single electron oxidation. In parallel to the mitigation of electrical losses, the catalyst in a certain medium defines the possible scenarios of postreactions and, therefore, the selectivity of the ORR toward different terminal products.

In this work, we synthesized mesoporous NiO and NiCo_2_O_4_ using a hydrothermal, template-free synthesis enabling control of the surface area and accessibility to active sites. The materials’ catalytic activity in ORR and OER was kinetically evaluated in half-cell measurements. Both catalysts were applied in ORR-based purification electrolyzers. Specifically, ORR on NiO was utilized in a hydroxyl radical generator, and ORR on NiCo_2_O_4_ was used in a device for oxygen-on-demand, i.e., an electrochemical oxygen purifier.

## 2 Experimental Section

### 2.1 Reagents

Nickel(II) nitrite hexahydrate, cobalt(II) nitrite hexahydrate, urea, potassium hydroxide, and coumarin were purchased from Merck (Sweden) and used as received. Ethanol and 2-proponal were purchased from Solvevo and used as received. Experiments were carried out with Milli-Q water from a Millipore Milli-Q system.

### 2.2 Synthesis

A new hydrothermal synthesis was used for the preparation of mesoporous nickel oxide and nickel cobaltite. Typically, 5 mmol metal salt (Ni/Co = 2:1 for NiCo_2_O_4_) and 22.5 mmol urea were dissolved in 228 mL Milli-Q water, and the mixture was stirred for 1 h to obtain a transparent solution. The solution was then transferred to a polytetrafluoroethylene (PTFE) bottle and placed in an oven for hydrothermal treatment (100 °C for 24 h). The resulting precipitation was collected by filtration and cleaned with water and ethanol several times. Mesoporous NiO and NiCo_2_O_4_ were obtained by calcining the precursor in a furnace at 400 °C for 2 h with a ramp of 5 °C min^–1^.

### 2.3 Physicochemical Methods

X-ray diffraction (XRD) was performed on a Panalytical X’Pert Pro X-ray diffractometer with Cu Kα radiation (λ = 0.15406 nm) and a Ni filter. Physisorption measurements were carried out on an ASAP 2020 (Micromeritics) at −196 °C using N_2_ as adsorbate. The samples were degassed at 300 °C for 6 h under a vacuum prior to analysis. The Brunauer–Emmett–Teller (BET) method was used to calculate the specific surface area of the materials at the pressure range *P*/*P*_0_ = 0.07–0.18, and the Barrett–Joyner–Halenda (BJH) method was used to calculate the pore size distribution and pore volumes based on the desorption branch of the isotherms. Transmission electron microscopy (TEM) and selected area electron diffraction (SAED) were performed with a FEI Tecnai G2 microscope operating at 200 kV. For TEM sample preparation, a few droplets of ink with the powder sample well dispersed in ethanol were placed on a Cu grid. SEM was carried out on a Sigma 300 operating at 5 kV. X-ray photoelectron spectroscopy (XPS) was conducted using a Kratos Axis Ultra DLD instrument equipped with monochromatic Al Kα radiation (*hv* = 1486.6 eV) and operating with an anode power of 150 W. The base pressure during analyses was below 1.1 × 10^–9^ Torr (1.5 × 10^–7^ Pa). All spectra were acquired at a normal emission angle and with a charge neutralizer. The analyzer pass energy was set to 20 eV, resulting in an energy resolution of 0.38 eV, as determined from the Fermi edge cutoff of reference Au and Ag samples. The calibration of the binding energy scale was confirmed by examining sputter-cleaned Au, Ag, and Cu samples (all in the form of polycrystalline thin films) according to the recommended ISO standards for monochromatic Al Kα sources that place Au 4f_7/2_, Ag 3d_5/2_, and Cu 2p_3/2_ peaks at 83.96, 368.21, and 932.62 eV, respectively.^24^ As the charge referencing based on adventitious carbon is not reliable,^25,26^ the spectra are presented as recorded, and interpretation is based on the shape of the spectral envelope rather than the binding energy values.

### 2.4 Electrochemical Measurements on Half-Cell Configuration

An SP-200/300 potentiostat (Bio Logic Science Instruments) was used for the electrochemical experiments. The measurements in half-cells were performed on a three-electrode setup using a glassy carbon electrode (GCE), platinum wire, and Hg/HgO as working, counter, and reference electrodes, respectively. The rotating (ring) disk electrode (R(R)DE, 5.61 mm OD, platinum ring 6.25 mm ID, 7.92 mm OD, Pine Research Instrumentation Inc.) was added to the three-electrode setup. Prior to use, the GCE was polished with a 0.05 μm Al_2_O_3_ suspension and sonicated in ethanol and water for cleaning.

In the ORR study, the number of transferred electrons per one oxygen molecule (*n*) and in situ H_2_O_2_ yield (Ψ_H_2_O_2__) can be estimated by the following equations:
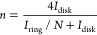
1
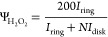
2where *I*_disk_ and *I*_ring_ are the currents recorded on the catalyst-modified disk and platinum ring, respectively, and *N* is the collection efficiency (0.32 for the RRDE utilized in this study).

The potential of the reference Hg/HgO electrode with respect to a reversible hydrogen electrode (RHE) was measured prior to the experiments. KOH (0.1 M) was saturated with H_2_ for 30 min, and the H_2_ flow was maintained in the electrolyte. Two Pt wires were used as working and counter electrodes, and Hg/HgO was used as the reference electrode. Linear sweep voltammetry was conducted at a scan rate of 5 mV s^–1^ from −1 to −0.8 V vs Hg/HgO, and the result (Figure S1) indicates that the potential of Hg/HgO is 0.890 V vs RHE. This was used to convert the potential scale to RHE in 0.1 M KOH by the Nernst equation: *E*_RHE_ = *E*_Hg/HgO_ + 0.089*E*_RHE_, where *E*_Hg/HgO_ and *E*_Hg/HgO_ are potentials measured with respect to RHE and Hg/HgO reference electrodes, respectively. The potential conversion for 1 M KOH takes the pH difference compared to 0.1 M KOH (0.059 × pH). Ohmic *E*_iR – corrected_ = *E*_RHE_ – *i* × *R*, where *E*_iR – corrected_ and *E*_RHE_ are corrected and converted potentials (V), *i* is the current (A), and *R* is the internal resistance (Ω) determined from electrochemical impedance spectrum (EIS) measurements.

### 2.5 Membrane Electrolyzers

Carbon fiber paper (CFP, Toray carbon paper 060, Fuelcellstore (USA)) was used as substrate for the catalysts. Prior to use, the CFP was calcined at 600 °C for 30 min to remove surface hydrophobic groups followed by washing in ethanol and water and drying at 80 °C overnight. The 5 mg mL^–1^ electrocatalyst ink was prepared by mixing 10 mg of the catalyst in 2 mL of a solution consisting of 980 mL of water, 980 mL of isopropyl alcohol, and 20 mL of 5 wt % Nafion solution in an ultrasound bath for 30 min. The catalyst ink was deposited on CFP by drop-casting with a loading of 1 mg cm^–2^ followed by drying in an oven at 80 °C overnight.

The flow membrane cell (C-flow 5 × 5 (active area of 25 cm^2^), C-Tech Innovation Ltd. (UK)) and anion-exchange membrane (AEM, Fumasep FAA3PKBO, FuelCellStore) were employed for assembling a device with a sandwiched structure. The graphite felt (AvCarb G200, FuelCellStore (TX, USA)) used as a diffusion layer was treated by immersing in concentrated H_2_SO_4_ followed by washing with flood water. Prior to use, the anion exchange membrane was immersed into 1 M KOH solution overnight to implement the exchange of Br and OH ions. The rate of flow of catholyte and anolyte is about 10 mL min^–1^.

#### 2.5.1 Electrochemical Generator of the Hydroxyl Radical

NiO- and NiCo_2_O_4_-modified CFPs were used as cathode and anode, respectively. The generator was assembled in a configuration of a flow cell. KOH (0.1 M) was fed as anolyte and catholyte. O_2_ flow (50 mL min^–1^ ) was maintained in the catholyte to enable oxygen saturation. Rhodamine B (RhB, 20 mg L^–1^) was fed into the cathode. Offline UV–vis measurements (300–600 nm and step size of 1 nm) were used for RhB quantification.

#### 2.5.2 Electrochemical Oxygen Purifier

NiCo_2_O_4_-modified CFPs were used as electrodes. KOH aqueous solutions (0.1–3 M) were used as anolyte and air-saturated catholyte. The generator was assembled in a flow cell configuration. The volume of produced oxygen on the anode after 2 h was collected and measured by a water displacement method.

## 3 Results and Discussion

### 3.1 Material Characterization

**Figure 1:**
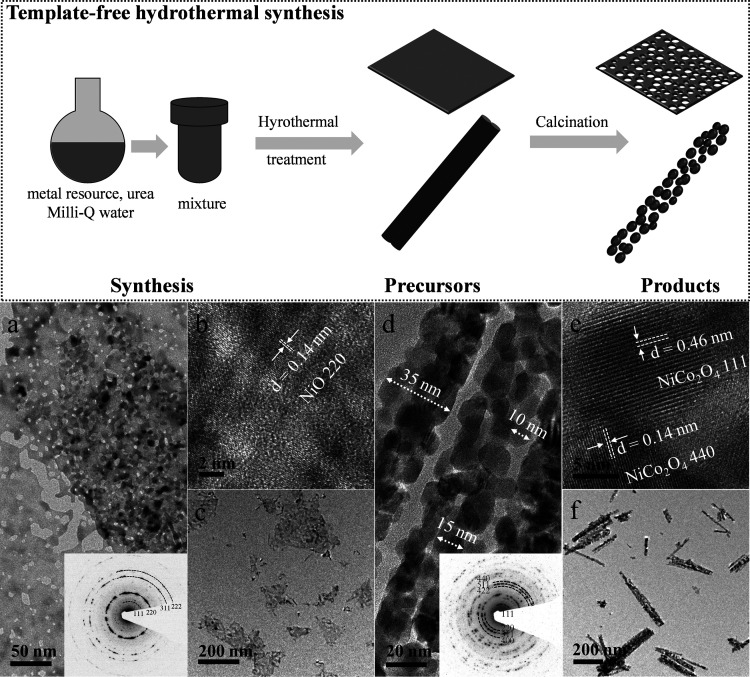
Top panel: scheme illustrating the synthesis process of mesoporous materials via a template-free hydrothermal route. Bottom panel: transmission electron micrographs with selected area electron diffractions as insets of mesoporous (a–c) NiO and (d–f) NiCo_2_O_4_.

**Figure 2:**
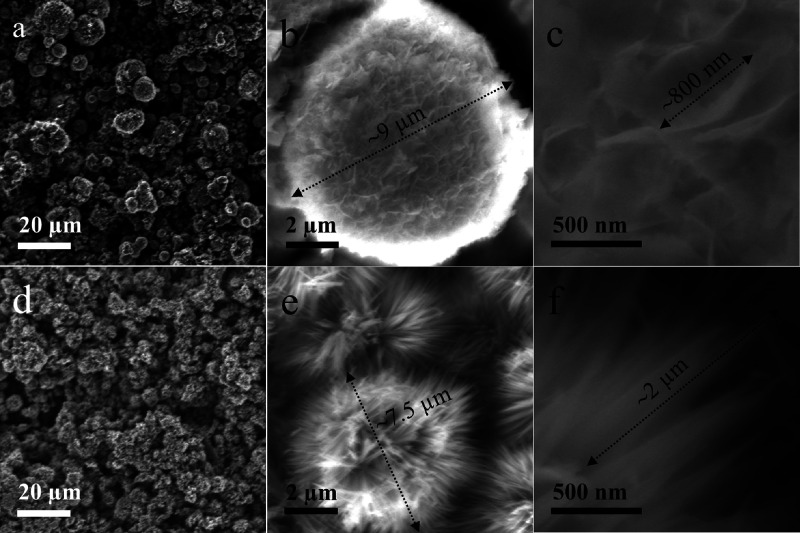
Scanning electron micrographs of mesoporous (a–c) NiO and (d–f) NiCo_2_O_4_.

A template-free hydrothermal synthesis route with urea as precipitant can generate abundant porous structures in crystalline materials due to the release of small molecules (i.e., carbon dioxide and water) during the thermal decomposition of hydrocarbonate precursors.^27−29^ The scheme in Figure 1 presents the synthesis process of mesoporous materials by a template-free hydrothermal route. Particularly, the metal resource is dissolved in Milli-Q water with urea that plays the role of precipitation agent by generating the OH group during the hydrothermal treatment over 80 °C.^27^ The release of small molecules of CO_2_ and H_2_O leads to the generation of abundant pores in target products during the transformation from metal hydrocarbonate precursors to products. Two different metal oxides, namely, mesoporous nickel oxide and nickel cobaltite, are accordingly prepared. Figure 1a,c presents the mesoporous NiO nanosheets, where the pores are observed at low-magnification TEM micrographs. The lattice fringe spacing (Figure 1b) corresponds to a d-spacing (*d*) of 0.14 nm and is assigned to 220 planes of nickel(II) oxide. NiCo_2_O_4_ (Figure 1d,f) consists of nanoneedles, about 35 nm wide, consisting of arrays of smaller particles, 10–15 nm in diameter with mesopores formed between them. The d-spacings observed in this material are 0.14 and 0.46 nm (Figure 1c), which correlate to the 440 and 111 planes of NiCo_2_O_4_. The selected area electron diffraction (SAED) patterns show bright rings that confirm the polycrystallinity of NiO and NiCo_2_O_4_, respectively. The morphology of mesoporous NiO and NiCo_2_O_4_ is further investigated by SEM as shown in Figure 2a–c and d–f, respectively. NiO and NiCo_2_O_4_ both show homogeneous nanosphere aggregates with different cross diameters of the particles. It is clear that NiO is composed of nanosheets with a width of about 800 nm, whereas NiCo_2_O_4_ consists of nanoneedles that have a length of 2 μm. This is in full agreement with the observations of TEM micrographs. The morphology differences between NiO and NiCo_2_O_4_ could be due to different reactivities of the metal ions used in the hydrolysis.^30^

**Figure 3:**
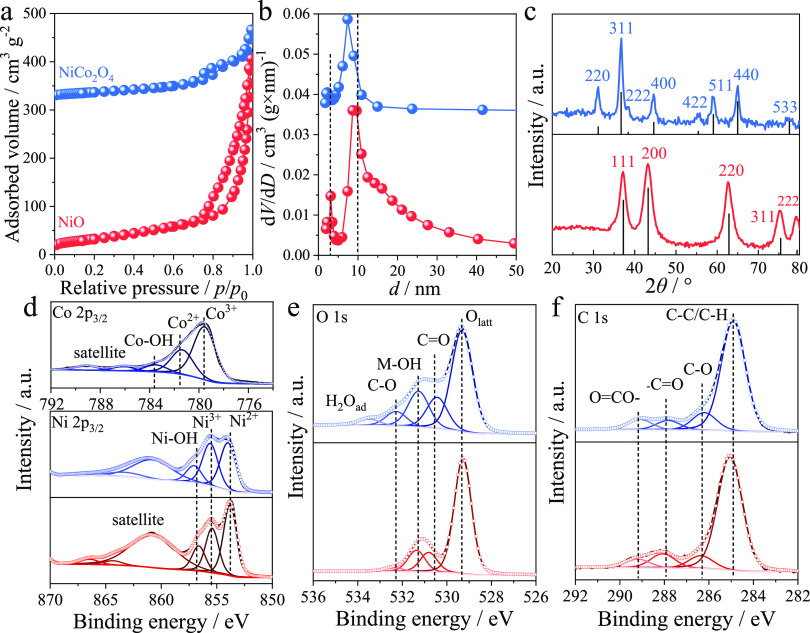
Physicochemical properties on mesoporous nickel(II) oxide and nickel cobaltite. (a) Physisorption isotherms, (b) pore size distributions, and (c) X-ray diffractograms. (d) Ni 2p_3/2_ and Co 2p_3/2_, (e) O 1s, and (f) C 1s high-resolution XPS spectra recorded from mesoporous NiO (red) and NiCo_2_O_4_ (blue) samples.

The pore characteristics and crystalline structure of the catalysts were studied by nitrogen physisorption and XRD, respectively. The physisorption of NiO and NiCo_2_O_4_ (Figure 3a) showed type IV isotherms with hysteresis loops typical for interparticle mesoporosity and intraparticle mesoporosity.^31^ The pore size distributions are narrow for both materials with a maximum of around 8–9 nm (Figure 3b, Table 1). The X-ray diffractograms (Figure 3c) show the cubic crystalline structure of NiO (#047-1049) and NiCo_2_O_4_ (#073-1702) without other phases detected. Specifically, in NiO, the diffraction peaks (Figure 3c, red curve) located at 2theta of 37.2, 43.2, 62.8, 75.4, and 79.4° are assigned to 111, 200, 220, 311, and 222 planes, respectively. The peaks in NiCo_2_O_4_ (Figure 3c, blue curve) at 31.1, 36.7, 38.3, 44.6, 55.4, 59.1, 64.9, and 77. 4° are correspondingly indexed to 220, 311, 222, 400, 422, 511, 440, and 533 planes. This agrees with the SAED patterns shown in Figure 3. The calculated crystal sizes (*D*’s) are ∼6.0 and 9.3 nm for NiO and NiCo_2_O_4_, respectively. For NiCo_2_O_4_, this is in good agreement with the microscopy data (∼10 nm). However, there is no agreement between the calculated crystal size for NiO and their sizes observed in TEM. This is due to the fact that the Scherrer equation analysis is based on spherical nanoparticles, which is not the case for sheet-shaped NiO.

**Table 1:** Physiochemical and Electrochemical Properties of Mesoporous NiO and NiCo_2_O_4_

	physicochemical properties							electrochemical properties				
	BET surface area (m^2^ g^–1^)	pore volume (cm^3^ g^–1^)	pore size (nm)	crystal size (*D*) (nm)				double-layer capacitance (*C*_dl_) (mF cm^–2^)	Tafel slope (mV dec^–1^)	η^OER^ (V)	*E*_onset_^ORR^ (V)	*n*_RRDE_
NiO	118	0.60	∼3, 9	6.0	0.22	0.22		1	56	0.38	0.65	3.2
NiCo_2_O_4_	61	0.20	∼2, 8	9.3	0.28	0.26	0.58	3	50	0.34	0.86	3.7

XPS Ni 2p_3/2_, Co 2p_3/2_, and O 1s core-level spectra recorded from mesoporous NiO and NiCo_2_O_4_ surfaces are shown in Figure 3d,e, respectively. The proposed peak fitting reveals three main distinctive peaks in both Ni 2p_3/2_ and Co 2p_3/2_ spectra, which are assigned to two different metal oxidation states, namely, 2+ and 3+, as well as to bonding with adsorbed hydroxyl species.^32,33^ Structures visible at high binding energy are attributed to the corresponding satellite peaks.^34^ The O 1s spectrum is fitted with three peaks assigned to the lattice oxygen (O_lattice_), adsorbed hydroxyl species (−OH), and the contaminants of carbonates.^35^ The adsorption of carbonates and water molecules on the surface is observed as peaks at higher binding energy. The presence of adventitious carbon is observed on the surface of both oxides. Deconvolution of the C 1s spectrum shows the presence of hydrocarbon, alcohol/ether, carbonyl, and ester depicted by four peaks from low to high binding energy (Figure 3f).^26,36^ The consistency observed between core-level Ni 2p_3/2_, Co 2p_3/2_, O 1s, and C 1s spectra enhances the reliability of the peak assignments. The close values of  and  ratios (namely, 0.22 and 0.22, and 0.26 and 0.28 for NiO and NiCo_2_O_4_, respectively, Table 1) imply the coherence in terms of hydroxyl-associated adsorbates on the surface.^37^ The atomic ratio of  calculated for NiCo_2_O_4_ materials is about 0.58 on the surface, suggesting the stoichiometric formation of nickel cobaltite with a ratio close to 0.50 that agrees with the X-ray diffractogram.

Conclusively, there is a good agreement in observations of microscopies, diffractograms, and physisorption results as well as surface chemistry results in terms of the porosity, crystalline structure, and compositions of the mesoporous NiO and NiCo_2_O_4_.

### 3.2 Electrochemical Half-Cell Measurements

**Figure 4:**
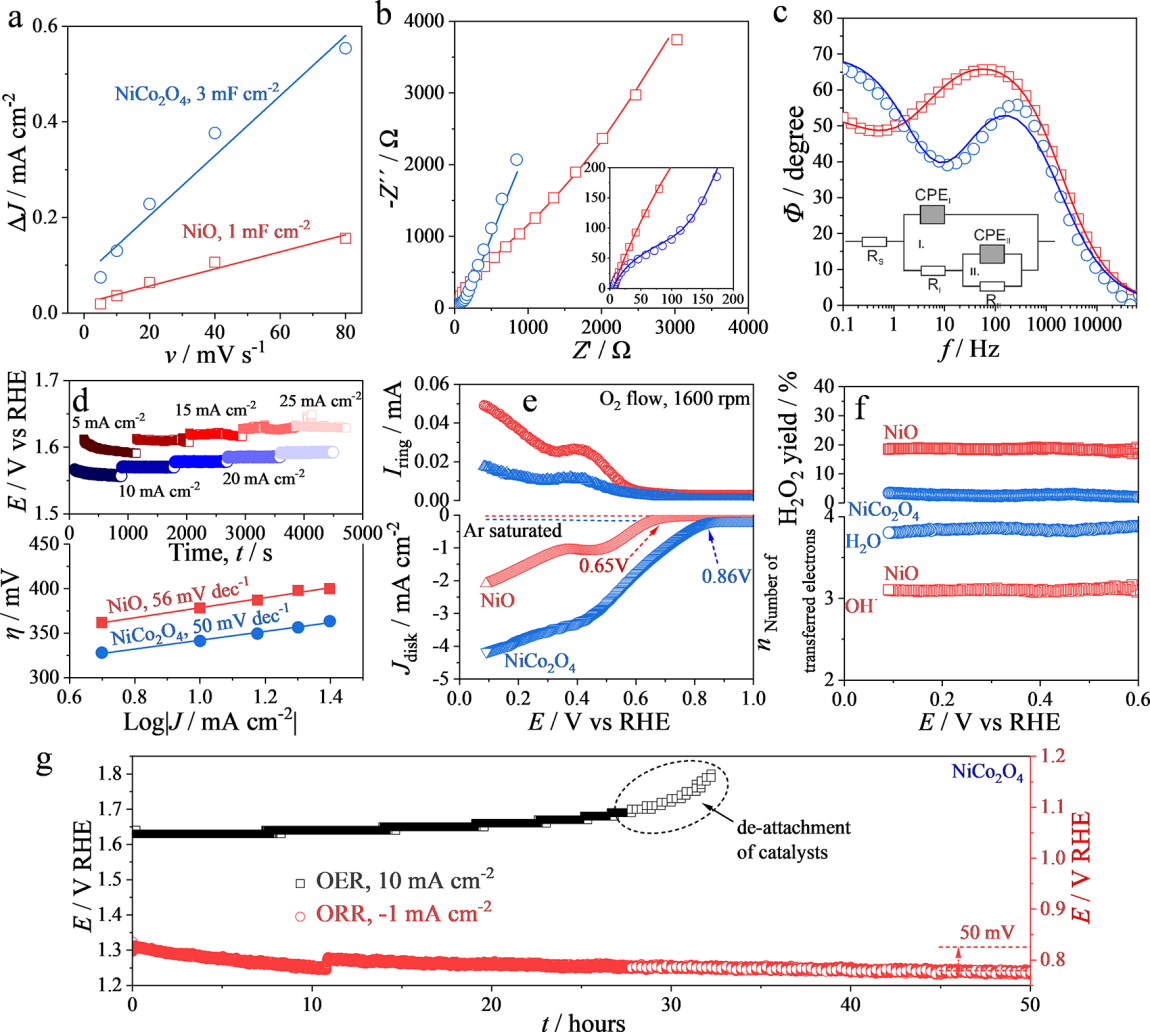
Half-cell measurements on mesoporous NiO- and NiCo_2_O_4_-modified electrodes with 0.1 mg cm^–2^ loadings (red and blue symbols, respectively). (a) The scan rate dependences of the capacitive currents. (b, c) The impedance spectra in Nyquist and Bode plots, respectively. The inset in panel c shows the equivalent circuit used for fitting of the impedance spectra. (d) Chronopotentiometry and reconstructed steady-state voltammogram of OER in Tafel coordinates (up and down, respectively; 1 M KOH, rotation speed 1600 rpm). (e) Linear sweep voltammograms recorded in ORR conditions on catalyst-modified rotating disk electrodes in argon- and oxygen-saturated 0.1 M KOH (rotation speed 1600 rpm, lower panel) and currents of H_2_O_2_ oxidation recorded on the platinum ring electrode (at potential 1.29 V vs RHE, upper panel). (f) The potential dependencies of in situ H_2_O_2_ yield (upper panel) and number of transferred electrons per oxygen molecule (lower panel). (g) Chronopotential voltammetry measurement of mesoporous NiCo_2_O_4_ for OER in 1 M KOH (10 mA cm^–2^) and ORR in 0.1 M KOH (−1 mA cm^–2^) at a rotating speed of 1600 rpm for up to 50 h.

The electrochemically active surface area, i.e., the catalyst interface exposed to the electrolyte solution and accessible for the formation of the electrical double layer, is visualized by the values of capacitive currents recorded on catalyst-coated electrodes (Figure S2). The specific surface area of NiO estimated by nitrogen physisorption is twice as large as for NiCo_2_O_4_ (Table 1). Oppositely, electrified NiO in contact with electrolyte solution showed a three times smaller electrochemically active surface area in comparison to NiCo_2_O_4_ of similar quantity (Figure 4a). Specifically, the capacitance of the electrical double layer formed on NiCo_2_O_4_ in contact with the electrolyte solution is significantly higher in comparison with that of NiO. This indicates a higher density of states on the NiCo_2_O_4_ surface, which could result in its higher capability for the heterogeneous electron transfer, i.e., catalysis of the ORR/OER.

Impedance spectroscopy was utilized for the assessment of electrochemical properties of porous catalysts (Figure 4b,c). The analysis of the spectra was based on the equivalent scheme comprising the solution ohmic resistance (Rs) and two R-C units. Considering the roughness of porous electrocatalysts, the constant phase elements were utilized during fitting instead of pure capacitors. A value of λ < 0.001 suggests a good fitting on spectra. The R-C constant calculated for R-C unit I is significantly lower than the value for R-C unit II (Table S1), indicating that process I is faster than process II. Therefore, process I is assigned to the charge transfer process in materials. Conclusively, an eight times smaller charge transfer resistance (*R*_I_, Table S1), which is reciprocal to the rate constant of the heterogeneous electron transfer, was observed on NiCo_2_O_4_ in comparison with NiO.

#### 3.2.1 OER

The electrode potentials required for OER were obtained by applying long-duration pulses (900 s) of various positive constant currents on the catalyst-coated working electrode (Figure 4d upper panel). This strategy allows both the exclusion of temporal nonequilibrium processes on the large catalytic interfaces and reconstruction of steady-state voltammograms of OER (Figure 4d upper panel).^38^ NiCo_2_O_4_ showed ca. 50 mV lower potentials in steady-state conditions compared to NiO. This manifests the mitigation of electrical energy loss by electrocatalysis on NiCo_2_O_4_. OER on both catalysts showed Tafel slopes close to 60 mV dec^–1^ (Figure 4d, lower panel), which can be assigned to an *EĈE* mechanism,^39,40^ where *E*’s are the preceding and subsequent electron transfers and *Ĉ* is a rate-determining chemical reaction. The slow chemical reaction could be deprotonation of chemosorbed hydroperoxyl: MOOH + OH^–^ ↔ MOO^–^ + H_2_O.^19^

#### 3.2.2 ORR

The launch of ORR on the catalyst-modified electrodes is visible by the significant increases of negative currents upon the introduction of dissolved oxygen in the electrolyte (Figure 4e lower panel). NiCo_2_O_4_ shows a higher ORR catalytic activity by both a higher onset potential (ca. 0.2 V) and two times higher current densities in comparison with the voltammetry data for the NiO-coated electrode. It has been reported that NiCo_2_O_4_ has a higher catalytic activity for oxygen associated reaction than either of the single metal oxides.^41^ DFT calculations by Zhao et al. show that hydroxyl modified bimetallic catalysts perform better than hydroxyl bonded single-metallic ones for both oxygen reduction and oxygen evolution reactions.^42^ The rotation of the catalyst-modified disk electrode fenced with an independent platinum ring electrode and biased by the potential of specific oxidation of H_2_O_2_ (so-called rotating ring disk electrode (RRDE)) creates a laminar flow of electrolyte solution. This operational setup enables the convective transport of intermediates appearing during the reaction from the catalyst-modified disk electrode to the ring electrode, which acts as an in situ intermediate detector. The positive currents of H_2_O_2_ oxidation on the platinum ring electrode are visible coherently with the appearance of negative currents of ORR on the catalyst-modified disk electrode. The NiO-modified electrode showed three times higher H_2_O_2_ oxidation currents compared to the NiCo_2_O_4_-modified electrode, indicating higher quantities of H_2_O_2_ formed during the ORR over NiO than over NiCo_2_O_4_. The lower hydrogen peroxide yield on NiCo_2_O_4_ can be attributed to a larger number of surface hydroxyl adsorbates that can promote the 4*e*-ORR path in comparison to the NiO.^43^

Quantitatively, the NiCo_2_O_4_-modified electrode (Figure 4f) in ORR conditions showed a higher number of transferred electrons (3.7)^43^ and lower H_2_O_2_ yield (ca. 5%) in comparison with the NiO-modified electrode (3.2 and ca. 20%, respectively). Considering the similar mass of the catalyst on the electrodes and the identical measurement conditions, it is possible to conclude that the alteration of the catalyst composition led to a selectivity change of ORR toward a terminal product. Specifically, ORR on NiCo_2_O_4_ proceeds to water as the terminal product bypassing desorbed H_2_O_2_, which is illustrated by the very low H_2_O_2_ yield and the number of transferred electrons close to 4 in coherence with a complete ORR-to-water:
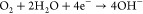
3

The results showing that NiCo_2_O_4_ has a 4*e*-ORR path with relatively low hydrogen peroxide are in good agreement with other previous works.^41,44,45^ The material has been applied in various applications like the positive electrode in Zn-air or lithium-oxygen batteries with specific metrics.^46,47^

On the other hand, ORR on NiO proceeds via the peroxo-pathway yielding desorbed H_2_O_2_, which is detected on the platinum ring electrode:
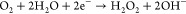
4Reaction 4 with H_2_O_2_ as the terminal product implies two transferred electrons, whereas the values observed in actual experiments are close to three. This discrepancy indicates that there is an additional ongoing monoelectron reduction process on the NiO-modified electrode in parallel to reaction 4. A Fenton-like reaction with H_2_O_2_ has been reported for nickel(II) species as a monoelectron reducer yielding nickel(III) and hydroxyl radical (reaction 5):^48^
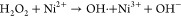
5Then, the monoelectron reduction process parallel to reaction 4 could be the electrochemical regeneration of Ni(II) species at the surface of NiO:
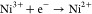
6which is fast because of the very high driving force (ca. 0.7 V difference between the ORR onset and equilibrium potential for Ni(OH)_2_/Ni(OH)_3_ redox couple in 0.1 M KOH).^49^Reaction 6 completes the catalytic cycle of reactions 4–6 as hydrogen peroxide reduction:^7,13^
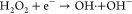
7The presence of the electrochemical regeneration of the Ni(II) species in the reaction cycle implies the generation of the hydroxyl radical via a Fenton-like reaction on the surface.

The presence of the hydroxyl radical during ORR on NiO was confirmed independently by offline fluorescence measurements.^50^ The fluorescence spectra of the electrolyte with additions of coumarin measured before and after 500 cycles of cyclic voltammetry showed three times higher intensity of the 7-hydroxycoumarin peak (510 nm), formed by coumarin oxidation by the hydroxyl radical, after cycling (Figure S3).

To consider the effects of roughness and mass loading, we compared the normalizations of OER and ORR kinetic currents (Table 2) either on the loaded mass or on the double-layer capacitance or BET surface area. In all comparisons, NiCo_2_O_4_ significantly outperforms mesoporous NiO in both OER and ORR, manifesting the genuine effect of the oxide composition on catalytic activity, which could be assigned to the distinctive characteristics, i.e., more abundant surface hydroxyl adsorbates, larger electrochemical capacitance, and smaller charger transferring resistance than NiO. In addition, the stability of mesoporous NiCo_2_O_4_ of OER and ORR is evaluated by the voltage loss by applying 10 and −0.1 mA cm^–2^ in realistic conditions, respectively (Figure 4g). For OER, the required potential increases gradually, with a significant increase after 30 h. This could be due to the detachment of the active materials as a result of O_2_ bubbles on the electrode surface. In ORR, the material shows a ∼ 50 mV voltage loss after 50 h. This confirms a high stability of the mesoporous NiCo_2_O_4_ for both oxygen reactions.

**Table 2:** Effect of Mesoporous Catalyst Characteristics on OER/ORR Kinetics

electrocatalyst	mass-normalized activity (A g^–1^)	activity normalized on capacitive currents (A F^–1^)	BET surface-normalized activity (A m^–2^)
OER, 1.58 V (RHE)			
NiO	51.02	5.10	0.84
NiCo_2_O_4_	255.10	8.50	2.16
ORR, 0.7 V (RHE)			
NiO	1.14	0.11	0.01
NiCo_2_O_4_	9.74	0.33	0.16

### 3.3 Membrane Electrolyzers

To utilize the demonstrated control of ORR selectivity by the catalyst composition, we assembled two model electrified purifiers to drive membrane electrolysis using two different cathode catalysts. An anion exchange membrane was used to maintain both the transport of hydroxide-anion associated with the ORR and OER on the cathode and anode and the barrier properties toward different chemical species associated with the electrode reactions. The mesoporous NiCo_2_O_4_ was used as OER anode catalyst in both electrolyzers due to its higher reactivity compared to the NiO.

#### 3.3.1 Electrochemical Generator of the Hydroxyl Radical

**Figure 5:**
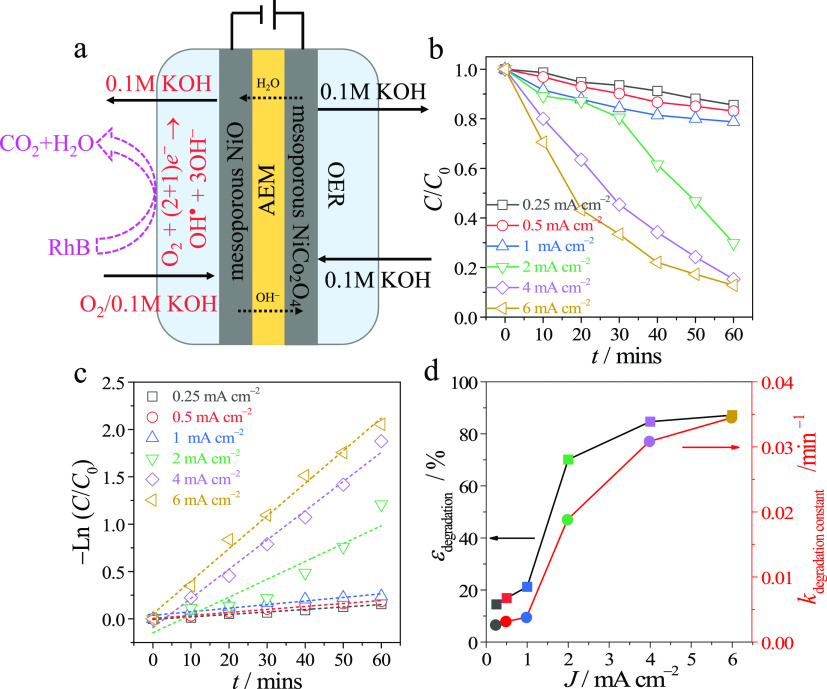
Electrochemical generator of the hydroxyl radical. (a) Scheme of operation of the hydroxyl radical generator. (b) The normalized concentration of RhB during the generator operation with different current densities. (c) The logarithm of normalized RhB concentration as a function of time. (d) The degradation efficiency and rate constant as a function of current density.

To generate the hydroxyl radical by ORR and prohibit its disappearance on the anode, NiO-modified CFP was used as a cathode in the membrane electrolyzer, fed by pure anolyte and oxygen-saturated catholyte (Figure 5a). RhB was used as a model organic contaminant and to observe the hydroxyl radical. The normalized intensity of RhB adsorption assayed by UV–vis decreases with increased electrolysis time (Figure 5b, Figure S4), manifesting the degradation of RhB due to the reaction with in situ-generated hydroxyl radical. Increasing the electrolysis current densities, the driving force of the process, led to the increase of the RhB degradation rate due to the increased production rate of the hydroxyl radical. Specifically, up to 90% of the initial RhB concentration was degraded in 1 h of operation at a current density of 6 mA cm^–2^ (Figure 5b, yellow curve). The linearity between the natural logarithm of degradation ratio and elapsed time suggests a first-order reaction of degradation by the hydroxyl radical (Figure 5c). The linear fitting of time dependencies showed the estimated rates of degradation of 0.003–0.034 min^–1^ at the different current densities with corresponding degradation efficiencies in a range of 10–90% (red and black curves in Figure 5d, respectively; Table S2). The operation cell voltage is lower than 2.4 V (Figure S5). To the best of our knowledge, this is the first demonstration of an electrochemical generator of the hydroxyl radical via controlled ORR to be used in organic pollutant degradation.

#### 3.3.2 Electrochemical Oxygen Purifier

**Figure 6:**
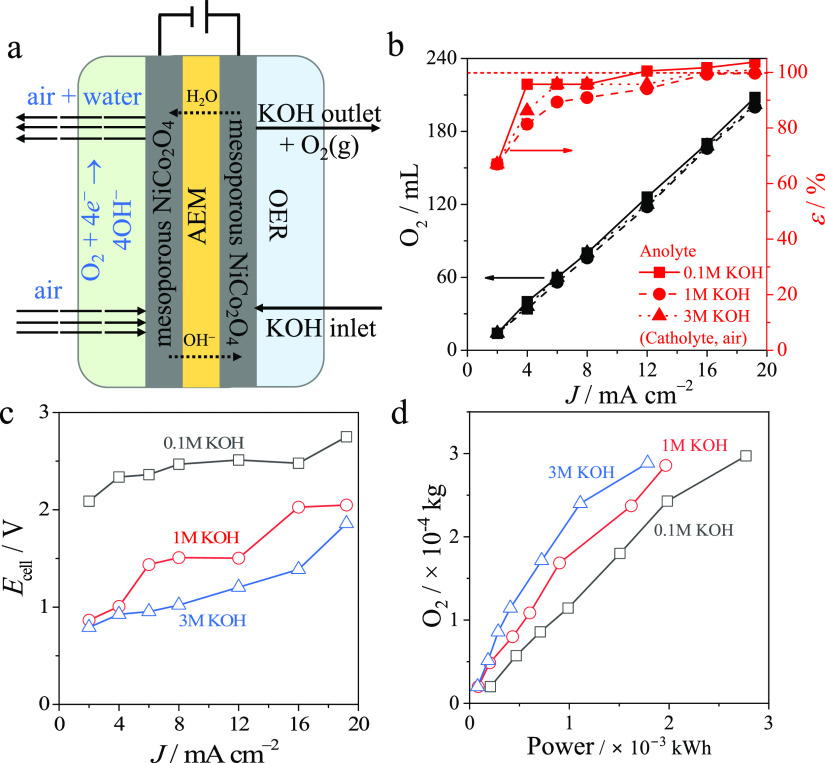
Electrochemical oxygen purifier. (a) Schematic of the oxygen purifier. (b) Generated oxygen volume and Faradaic efficiency as functions of the electrolysis current density (black and red symbols, respectively). (c) The dependencies of cell voltage on the electrolysis current density for different anolyte concentrations. (d) The generated oxygen volume as a function of the working power of the electrochemical cell with different anolyte concentrations.

A symmetrical membrane electrolyzer was assembled using an anion-exchange membrane sandwiched between two NiCo_2_O_4_-modified CFP as ORR cathode fed with air and oxygen-producing OER anode fed with KOH (Figure 6a). The volume of produced oxygen during 2 h of operation increases with the applied current (Figure 6b), illustrating that no air leaks into the synthesized oxygen. The oxygen production rate was independent of the anolyte concentration. This manifests the fast electrolyte-insensitive kinetics of the overall Faradaic reaction: 4OH^–^ → 4e^–^ + O_2_ + 2H_2_O. Oppositely, increasing the anolyte concentration led to a visible mitigation of the electrical energy loss represented by the operational cell voltages and the working power (Figure 6c,d). The ohmic resistance of the electrolyzer is independent of the anolyte concentration as estimated at the zero current conditions by high-frequency measurements (Figure S6). Therefore, the only process that remains affected by the increase of the anolyte concentration is the ionic currents across the membrane. The excess of the cations in the outer electrolyte solution in comparison with the quantity of immobile cations in the bulk of the anion-exchange membrane leads to increased kinetics of the ion transport across the membrane and the mitigation of the associated loss of the electrical energy.

The efficiency of the oxygen purifier was estimated by the ratio between the charge spent on the formation of the certain amount of oxygen and the inputted electrical charge using the following equation:
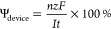
8where Ψ_device_ is the Faradaic efficiency of the oxygen purifier, *z* is the number of transferred electrons per oxygen molecule (4), *F* is the Faraday constant (96,485 C mol^–1^), *I* is the current of electrolysis (A), *t* is the time (s), and *n* is the amount of moles of produced oxygen estimated by , in which *V*_gas_ and *V*_STP_ are the volume of collected gas and molar volume of ideal gas at standard temperature and pressure conditions (22.4 L mol^–1^). The Faradaic efficiency increases with increasing electrolysis current (Figure 6b), illustrating the mitigation of the operational losses. Importantly, the values of Faradaic efficiency exceed 100%, which illustrate the proceeding of parallel gas-producing processes such as the oxidation of graphite to carbon dioxide at high anodic potentials^51^ and the need of further optimization. The effect is slightly more pronounced in the 0.1 M KOH condition compared to 1–3 M KOH, as observed from the slightly higher Faradaic efficiency. This could be due to the fact that a higher cell voltage over the device leads to more severe corrosion of the cabron component. Mesoporous NiCo_2_O_4_ materials coated on CFP after the measurement are characterized with SEM and XPS, which show that the NiCo_2_O_4_ kept the nanoneedle morphology despite the changes of nanospherical aggregates due to the mixture with organic binders and the ultrasonic treatment during the preparation of the electrode (Figure S7). The shape analysis on the XPS spectrum shows the maintaining of different oxidation states of cations (2+, 3+) and the presence of hydroxyl adsorbates on the surface after the measurement (Figure S8), which suggest the stability of the material.

Decisively, the operational cell voltage of the oxygen purifier obtained in the optimized anolyte concentration (0.804 V) is lower than the Nernst limit of water electrolyzers (1.23 V). This implies that the production by the oxygen purifier is more efficient from the perspective of electrical losses and safety compared to the production of oxygen as a side product of green hydrogen technologies. The high operational stability of the oxygen purifier in combination with cost-effective catalysts is illustrated by the stable operational cell voltage (Figure S9).

## 4 Conclusions

The composition and structure of bifunctional ORR/OER catalysts NiO and NiCo_2_O_4_ were controlled using a hydrothermal synthesis in aqueous media. The template-free synthesis route resulted in mesoporous NiO and NiCo_2_O_4_ with specific hydroxyl adsorbates. The kinetics and the selectivity of both ORR and OER were evaluated on the catalysts in alkaline media. Mesoporous NiCo_2_O_4_ has a more efficient bifunctional oxygen activity compared to NiO shown by the higher normalized oxygen catalytic activity. The alteration of ORR product selectivity either to water on NiCo_2_O_4_ or to hydroxyl radical on NiO was observed in half-cell measurements. The ORR-to-hydroxyl radical path established on NiO was explored in an aqueous flow purifier based on the in situ generation of the hydroxyl radical. This approach is relevant for the technologies of electrified decontamination of water. Also, the ORR-to-water path established on NiCo_2_O_4_ was utilized in the construction of a high-efficiency oxygen purifier. The critical role of the electrolyte was demonstrated in the optimization of the device efficiency.

## Supporting Information Available

Measurement of the potential of the Hg/HgO reference electrode, CVs of mesoporous NiO and NiCo_2_O_4_ at different scan rates in the non-Faradaic region, parameters derived from EIS fitting, fluorescence measurement of electrolyte for hydroxyl-coumarin, RhB degradation rates with various different currents applied on the OH· generator, recorded cell voltage over the hydroxyl radical generator, SEM micrographs and XPS spectrum of NiCo_2_O_4_ after the full-cell measurement on electrochemical oxygen purifier, recorded EIS spectra, and the recorded cell voltage over the electrochemical oxygen purifier at various electrolytes (PDF)
