# Supplementary material for: Selectivity Control of Oxygen Reduction Reaction over Mesoporous Transition Metal Oxide Catalysts for Electrified Purification Technologies
Source: ACS Appl Mater Interfaces. 2023 May 19;15(21):26093–103. doi: 10.1021/acsami.3c01196 (PMC10236433; doi:10.1021/acsami.3c01196)
Supplement: Supplementary file 1 — am3c01196_si_001.pdf [file am3c01196_si_001.pdf]

# *Supporting Information*

# Selectivity Control of Oxygen Reduction Reaction over Mesoporous Transition Metal Oxide Catalysts for Electrified Purification Technologies

*Zhixing Wu[1*], Mikhail Vagin[2], Robert Boyd[1], Penghui Ding[2], Oleksandr Pshyk[3], Grzegorz Greczynski[3], Magnus Odén[1], and Emma M. Björk[1]*

1 Nanostructured Materials, Department of Physics, Chemistry and Biology (IFM), Linköping University, Linköping SE 58183, Sweden

2 Laboratory of Organic Electronics, Department of Science and Technology (ITN), Linköping University, Norrköping SE 60174, Sweden

3 Thin Film Physics, Department of Physics, Chemistry and Biology (IFM), Linköping University, Linköping SE 58183, Sweden

** Corresponding author (Zhixing Wu). Tel: +46 13-28 25 42, Email: zhixing.wu@liu.se*

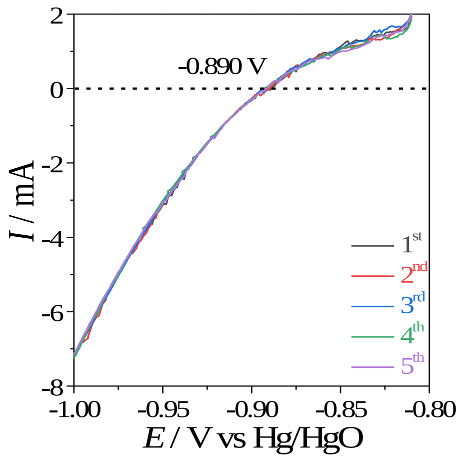


**Figure S1.** Linear sweep voltammograms of three-electrode set up using two Pt wires as working and counter electrodes, and Hg/HgO as reference electrode; the measurements were conducted in 0.1 M KOH with flowing $H_2$ at scan rate of 5 mV $s^{-1}$.

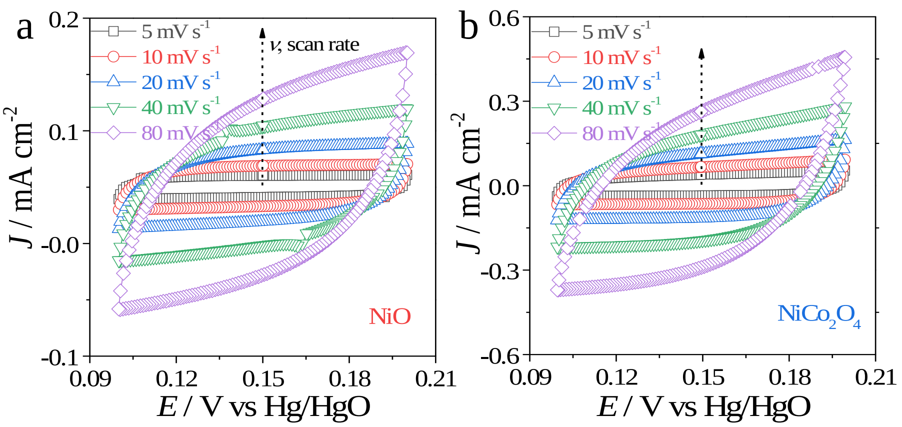


**Figure S2.** Cyclic voltammograms obtained with scan rates of 5 – 80 mV $s^{-1}$ on mesoporous (a) NiO and (b) $NiCo_2O_4$.

**Table S1.** Set of parameters derived from fitting of EIS data.

| Samples | $R_S$ / Ω | $P_{CPE\ I}$ / × $10^{-5}$ | $\phi_{CPEI}$ | [a]$C_I$ / × $10^{-5}$ F | $R_I$ / Ω | [b]$RC_I$ / ms | $P_{CPE\ II}$ / × $10^{-5}$ | $\phi_{CPE\ I}$ | [c]$C_{II}$ / × $10^{-5}$ F | $R_{II}$ / × $10^{5}$ Ω | [d]$RC_{II}$ / ms |
|---|---|---|---|---|---|---|---|---|---|---|---|
| **Mesoporous NiO** | 5.56 | 9.18 | 0.80 | 1.38 | 1544 | 0.021 | 23.44 | 0.59 | 0.25 | 9.92 | 2.47 |
| **Mesoporous $NiCo_2O_4$** | 5.27 | 15.60 | 0.77 | 1.83 | 190 | 0.003 | 50.00 | 0.80 | 11.37 | 1.62 | 18.42 |

[a] Estimated as: $C_I = \left(P_I \times (R_S)^{(1-\phi_I)}\right)^{1/\phi_I}$

[b] $RC1 = R_I \times C_I$

[c] $C_{II} = \left(P_{II} \times (R_S)^{(1-\phi_{II})}\right)^{1/\phi_{II}}$

[d] $RC2 = R_{II} \times C_{II}$

where $R_s$ is the solution resistance, P is a fitting parameter of the CPE and $\phi$ is the fitted exponent factor, which varies from 0 to 1. When $\phi$ is tending to 0 the CPE behaves as a pure resistor, and when $\phi$ is tending to 1 the CPE represents a pure capacitor.

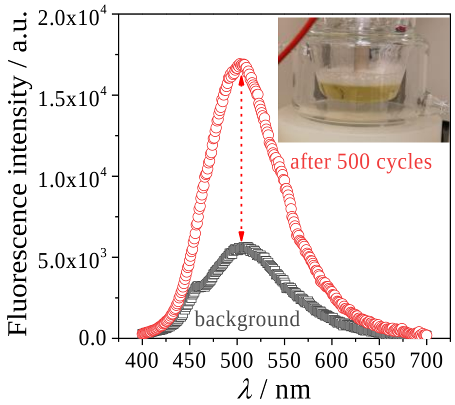


**Figure S3.** Fluorescence measurements of the electrolyte before and after 500 cyclic voltammetry measurements (black square and red circle, respectively) on mesoporous NiO modified glassy carbon electrode (GCE) in 0.1 M KOH. The inset shows a photo of the electrolyte after 500 cycles with a brownish color indicating oxidation of coumarin to 7-OH-coumarin by $OH^{\bullet}$.

**Table S2.** The RhB degradation rate obtained using the hydroxyl radical generator with various currents.

| **Currents (mA cm$^{-2}$)** | **Degradation rate (min$^{-1}$)** |
|---|---|
| **0.25** | 0.003 |
| **0.5** | 0.003 |
| **1** | 0.004 |
| **2** | 0.019 |
| **4** | 0.031 |
| **6** | 0.034 |

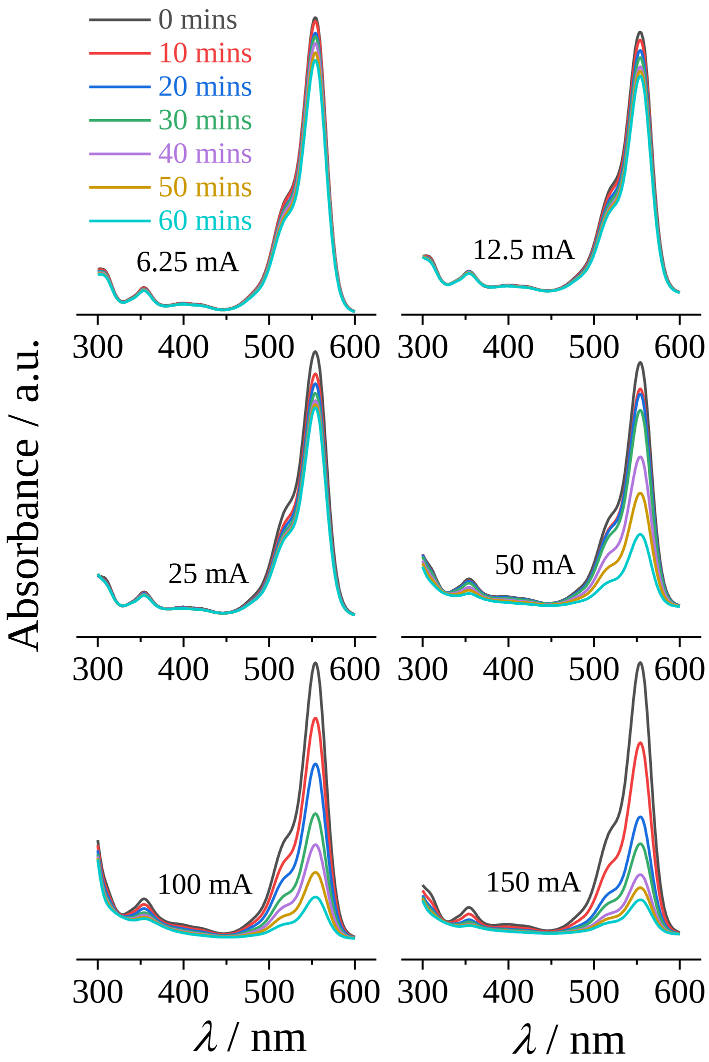


**Figure S4.** UV-vis spectra of RhB during the in situ degradation by $OH^{\bullet}$ obtained by the hydroxyl radical generator operated with currents of 6.25 – 150 mA.

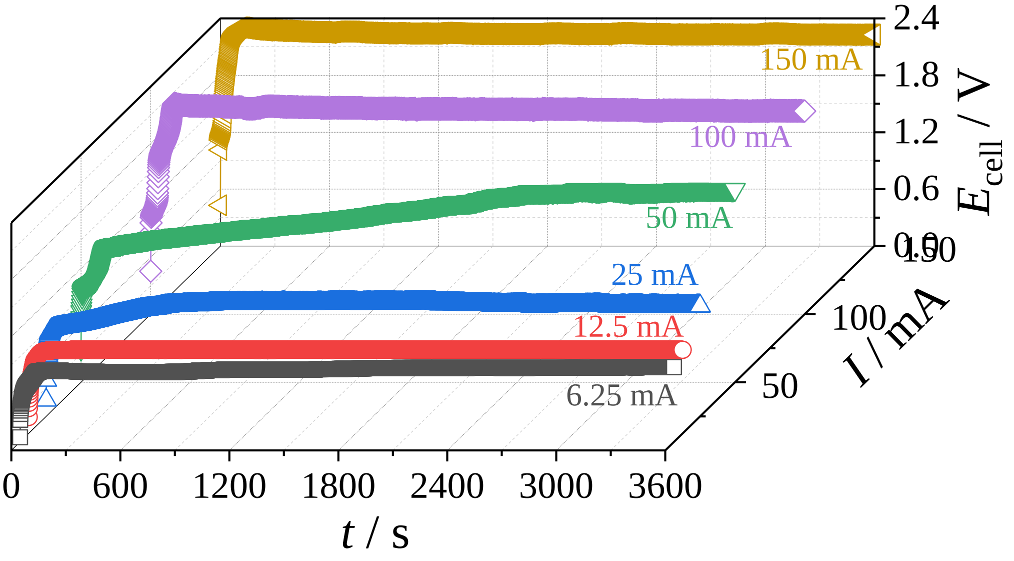


**Figure S5.** The recorded cell voltage as a function of time and applied current of 6.25 – 150 mA over the hydroxyl radical electrochemical generator.

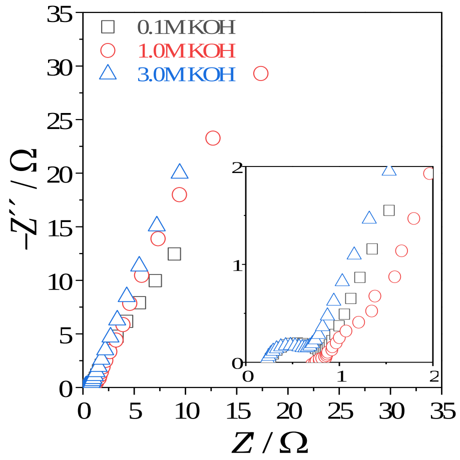


**Figure S6.** EIS spectra obtained from the electrochemical oxygen purifier with different anolyte concentrations at open circuit potential.

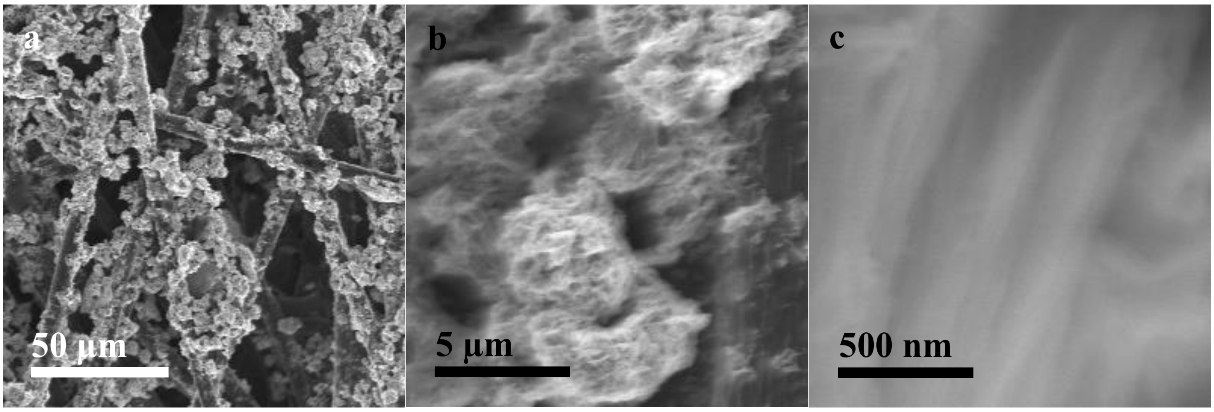


**Figure S7.** (a-c) Scanning electron micrographs of mesoporous $NiCo_2O_4$ coated on CFP as anode after measurement of electrochemical oxygen purifier.

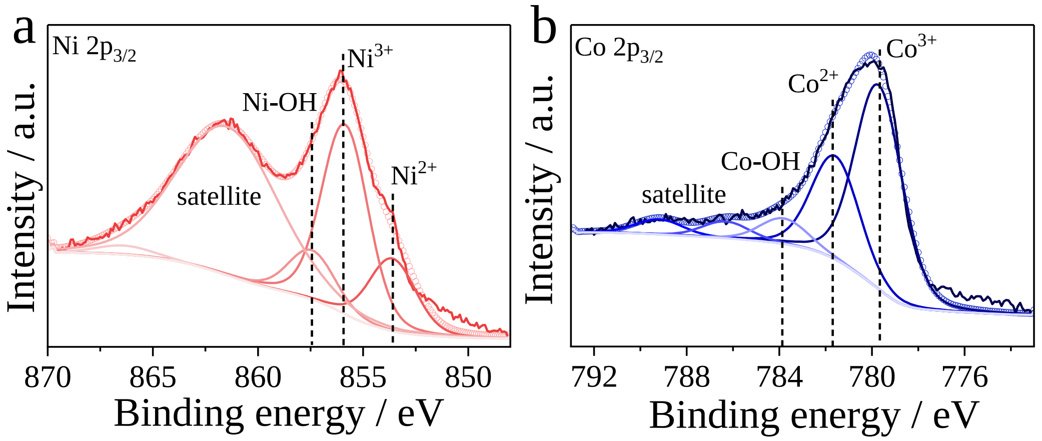


**Figure S8.** (a) Ni $2p_{3/2}$, and (b) Co $2p_{3/2}$ high resolution XPS spectra recorded from mesoporous $NiCo_2O_4$ coated on CFP as anode after measurement of electrochemical oxygen purifier.

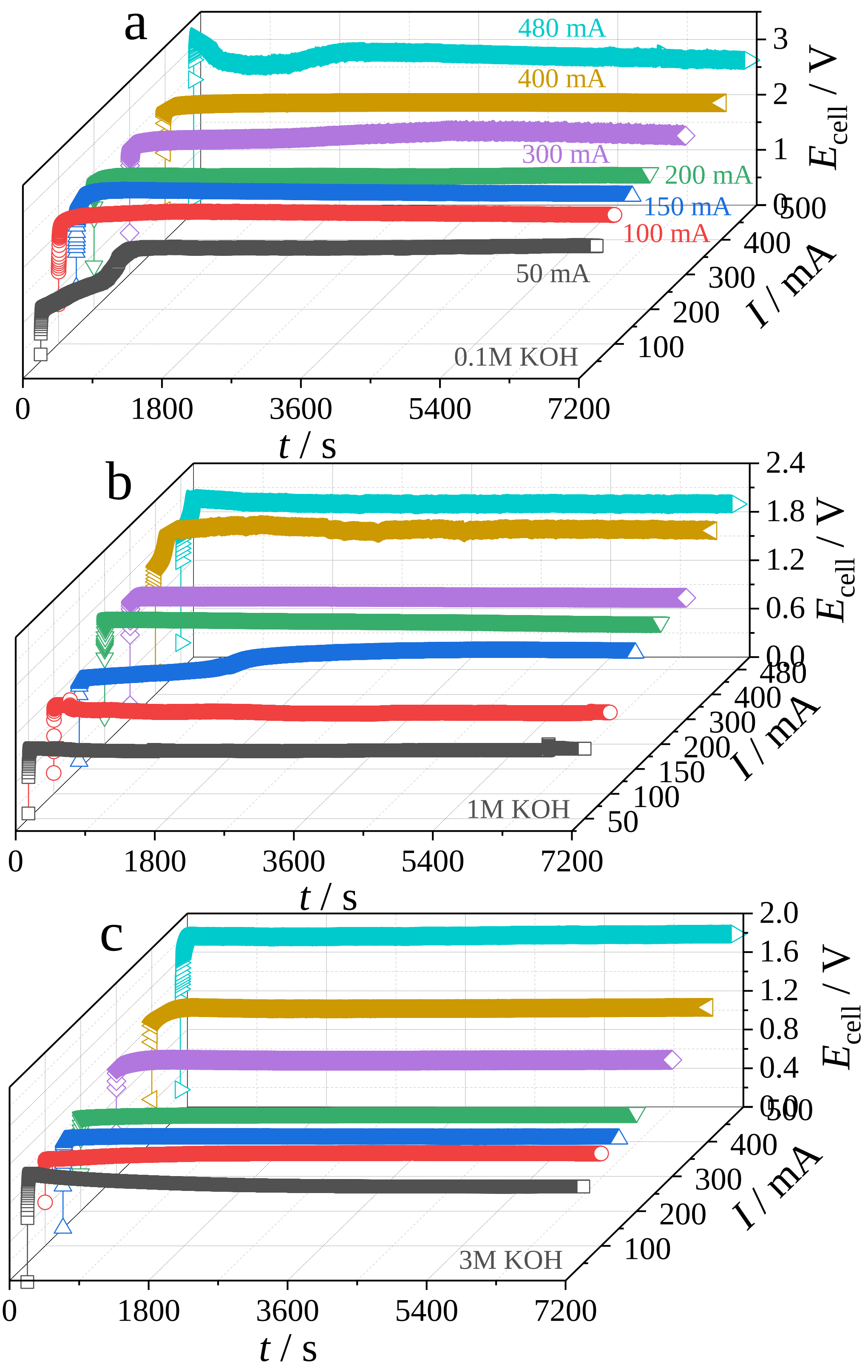


**Figure S9.** The recorded cell voltage as a function of time and applied currents of 50 – 480 mA over electrochemical oxygen purifier with a feed of (a) 0.1 M KOH, (b) 1 M KOH, and (c) 3 M KOH.
